# Association between PPARGC1A polymorphisms and the occurrence of nonalcoholic fatty liver disease (NAFLD)

**DOI:** 10.1186/1471-230X-8-27

**Published:** 2008-06-27

**Authors:** Masato Yoneda, Kikuko Hotta, Yuichi Nozaki, Hiroki Endo, Takashi Uchiyama, Hironori Mawatari, Hiroshi Iida, Shingo Kato, Kunihiro Hosono, Koji Fujita, Kyoko Yoneda, Hirokazu Takahashi, Hiroyuki Kirikoshi, Noritoshi Kobayashi, Masahiko Inamori, Yasunobu Abe, Kensuke Kubota, Satoru Saito, Shiro Maeyama, Koichiro Wada, Atsushi Nakajima

**Affiliations:** 1 Division of Gastroenterology, Yokohama City University Graduate School of Medicine, 3-9 Fuku-ura, Yokohama, Japan; 2 Laboratory for Endocrinology and Metabolism, Center for Genomic Medicine, RIKEN, 1-7-22 Suehiro, Tsurumi-ku, Yokohama, Japan; 3 Kitakashiwa Rehabilitation Hospital, 265 Kashiwasita, Kashiwa, Japan; 4 Department of Pharmacology, Osaka University, Graduate School of Dentistry, 1-8 Yamadaoka, Suita, Osaka, Japan

## Abstract

**Background:**

Genetic factors as well as environmental factors are important in the development of NAFLD and in this study we investigated associations between polymorphisms of peroxisome proliferators-activated receptor γ coactivator 1α polymorphism (*PPARGC1A*) and NAFLD.

**Aims:**

We recruited 115 patients with biopsy-proven NAFLD, 65 with NASH and 50 with simple steatosis, and 441 healthy control subjects and investigated 15 SNPs of *PPARGC1A*.

**Results:**

SNP rs2290602 had the lowest *p *value in the dominant mode (*p *= 0.00095), and the odds ratio for NAFLD (95% CI) was 2.73 (1.48 – 5.06). rs2290602 was significantly associated with NAFLD even when the most conservative Bonferroni's correction was applied (*p *= 0.0143). The frequency of the T allele of rs2290602 was significantly higher in the NASH patients than in the control subjects (*p *= 0.00093, allele frequency mode), and its frequency in the NASH patients tended to be higher than in the simple steatosis patients (*p *= 0.09). The results of the real-time RT-PCR study showed that intrahepatic mRNA expression of *PPARGC1A *was lower in the TT group than in the GG or GT group at SNP rs2290602 (p = 0.0454).

**Conclusion:**

This is the first study to demonstrate a significant association between genetic variations in *PPARGC1A *and NAFLD. This finding suggested that *PPARGC1A *polymorphism and lower expression of *PPARGC1A *mRNA in the liver are an important genetic contribution to etiology of NAFLD.

## Background

Nonalcoholic fatty liver disease (NAFLD) is one of the most common causes of chronic liver injury in many countries in the world [1,2], and epidemiological studies have shown that its prevalence ranges from 17% to 33% of the general population. NAFLD represents a spectrum of conditions that are histologically characterized by macrovesicular hepatic steatosis, and the diagnosis is made in patients who have not consumed alcohol in amounts sufficient to be considered to be harmful to the liver. The histological changes range over a wide spectrum, extending from simple steatosis, which is generally non-progressive, to nonalcoholic steatohepatitis (NASH), liver cirrhosis, liver failure, and sometimes even hepatocellular carcinoma [3,4].

Genetic factors as well as environmental factors are important to the development of NAFLD [5-8], and the gene for peroxisome proliferator-activated receptor γ coactivator 1α (*PPARGC1A*) is a candidate gene for susceptibility to NAFLD, since it is involved in insulin resistance, mitochondrial biogenesis, and oxidative phosphorylation, which are key factors in the development of NAFLD [9-11]. Recent evidence also implicates *PPARGC1A *in the homeostatic control of systemic energy metabolism, and *PPARGC1A *knockout mice have been reported to develop hepatic steatosis due to a combination of reduced mitochondrial respiratory capacity and increased expression of lipogenic genes [12]. Single nucleotide polymorphisms (SNPs) are useful tools in the search for genetic factors responsible for disease and are being intensively investigated in various common diseases, such as obesity, diabetes, and hypertension. Expression of *PPARGC1A *has been reported to be associated with metabolic factors, such as type 2 diabetes, hypertension, obesity [13-18].

In this study we investigated associations between SNPs of *PPARGC1A *and NAFLD in the Japanese subjects.

## Methods

### Subjects

A total of 115 Japanese NAFLD patients, 65 with NASH and 50 with simple steatosis, and 441 healthy control subjects were recruited to participate in this study at Yokohama City University Hospital. All control subjects were confirmed to have normal liver function, not to have viral hepatitis, and not to be alcoholics. The control subjects all had a BMI < 25 kg/m^2^, normal fasting glucose (<110 mg/dl), serum triglycerides (<150 mg/dl), and serum HDL cholesterol (>40 mg/dl) levels, and normal systolic (<130 mmHg) and diastolic blood pressure (<85 mmHg). Liver biopsy was performed in all 115 NAFLD patients, and the liver biopsy tissue obtained was stained with hematoxylin-eosin, reticulin stain, and Masson trichrome stain. The histological criterion used to make the diagnosis of NAFLD was the presence of macrovesicular fatty change in hepatocytes with displacement of the nucleus to the edge of the cell [19]. When more than 5% of hepatocytes were affected by macrovesicular steatosis, the patient was diagnosed as having either steatosis or steatohepatitis. The criteria used to make the diagnosis of steatohepatitis were the presence of lobular inflammation and the presence of either ballooning cells or perisinusoidal/pericellular fibrosis in zone 3 of the hepatic acinus, in addition to steatosis [20,21]. Patients with any of the following diseases were excluded from participation in this study: infectious hepatitis (chronic hepatitis C infection or concurrent active hepatitis B virus), autoimmune hepatitis, primary biliary cirrhosis (PBC), sclerosing cholangitis, hemochromatosis, α1-antitrypsin deficiency, Wilson's disease, drug-induced hepatitis, and alcoholic hepatitis, and heavy alcohol consumers (current or past daily consumption of more than 20 g alcohol per day). No patients had clinical evidence of hepatic decompensation, such as hepatic encephalopathy, ascites, variceal bleeding, or a serum billirubin level greater than twice the upper limit of normal.

Written informed consent was obtained from all subjects before their entry into this study. The study protocol conformed to the ethical guidelines of the 1975 Declaration of Helsinki and was approved by the Research Committee of Yokohama City Hospital.

### Physical and laboratory evaluation

The body weight and height of the patients were measured with a calibrated scale after requesting them to remove their shoes and any heavy clothing. A venous blood sample was obtained from the patients after an overnight fast (12 hours) to measure their serum AST, ALT, glucose, immunoreactive insulin (IRI), hemoglobin A1c (HbA1c), total cholesterol, HDL cholesterol, and triglyceride levels. All laboratory biochemical parameters were measured with a conventional automated analyzer. Visceral fat area (VFA) and subcutaneous fat area (SFA) were measured by computed tomography (CT).

### DNA preparation and SNP genotyping

Genomic DNA was prepared from each blood sample by using a commercial genomic DNA extraction kit (TALENT s.r.l., Trieste, Italy). The *PPARGC1A *SNPs were selected from the IMS-JST (Institute of Medical Science-Japan Science and Technology Agency) SNP database [22]. We selected the 15 SNPs with a minor allele frequency greater than 0.2 and whose expected allele frequencies did not widely diverge from Hardy-Weinberg equilibrium (*p *> 0.001). Invader probes (Third Wave Technologies, Madison, WI) were synthesized for these SNPs, and the SNPs were genotyped in the cases and controls by a combination of multiplex PCR and the Invader assay, as described previously [23].

### Real-time RT-PCR for measurement of PPARGCA1 mRNA expression

Total RNA was isolated from samples of liver biopsy specimens by using an RNeasy Mini Kit (Quiagen, Hilden, Germany) according to the manufacturer's instructions. The protocol included a DNase treatment step to remove genomic DNA. The RNA was assessed quantitatively by measuring relative absorbance at 260 nm and 280 nm, and qualitatively by ethidium bromide agarose-gel electrophoresis. Reverse transcription to produce cDNA was performed by using a TaqMan Reverse Transcription Reagents (Applied Biosystems, Foster City, CA, USA), according to the manufacturer's instructions. The reaction mixtures (100 μl) contained 2.5 μg of total RNA, and after allowing the reaction to proceed for 50 minutes at 48°C, the reverse transcriptase was inactivated by heating the samples to 95°C for 5 minutes.

Real-time quantitative RT-PCR was performed in triplicate by using an ABI Prism 7700 sequence detection system (Applied Biosystems, Foster City, CA, USA) and SYBR Green PCR Master Mix according to the manufacturer's protocol. The following primers were used: *PPARGC1A *(F, 5'-TCTGACGTGACCATGGTGTT-3'; R, 5'-CATTCCAGGGACTCCACACT-3'). The primers were designed with Primer Express software (Applied Biosystems, Foster City, CA, USA) based on the sequence data obtained from the GenBank database. β-actin (Applied Biosystems, Foster City, CA, USA) was used as a reference; i.e., each sample was normalized on the basis of its β-actin content. Thermal cycling was performed as follows: initial denaturation at 95°C for 10 minutes, followed by 40 cycles of 95°C for 15seconds and 60°C for 1 minute.

### Statistical analysis

For each case-control study, the frequencies of the genotypes or the alleles were compared between cases and controls in three different modes by means of the χ^2 ^test. In the first mode (allele frequency mode), allele frequencies were compared between cases and controls by means of a 2 × 2 contingency table. In the second mode (recessive mode), the frequencies of the subjects who were homozygous for allele 1 were compared with the rest by means of a 2 × 2 contingency table, while in the third mode (dominant mode) the frequencies of the subjects who had allele 1 (allele 1 homozygotes and heterozygotes) were compared with the rest by means of a 2 × 2 contingency table. The odds ratio (OR) and its 95% confidence interval (CI) were calculated by Woolf's method. Conformity to the Hardy-Weinberg equilibrium was assessed by the χ^2 ^test [24]. Haplotype blocks were calculated using Haploview 3.2 software [25]. Physical and laboratory data are reported as means ± standard deviation (SD).

## Results

### Case-control association study

**Figure 1:**
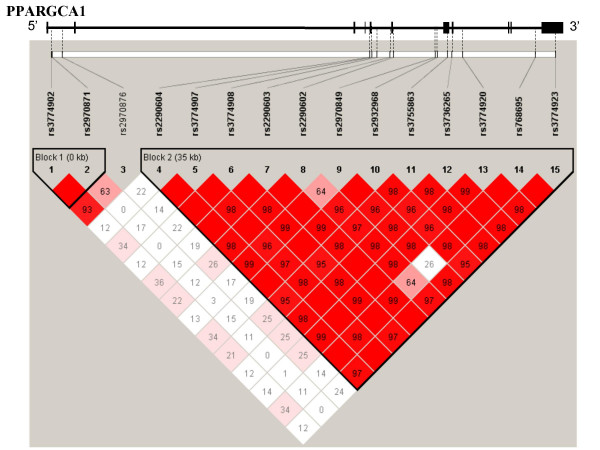
**LD mapping around the PPARGC1A gene**. LD coefficients (d) were calculated for every pair of *PPARGC1A *SNPs. LD coefficients (D') were calculated for every pair of SNPs and are shown as the strand of the LD blocks. The minor allele frequencies of all SNPs used in this analysis were >20%. Each SNP is labeled with its rs number.

The characteristics of NAFLD groups and control group are compared in Table 1. We selected the 15 SNPs of the *PPARG1A *gene in the IMS-JST SNP database that had a minor allele frequency greater than 0.2. The information on SNP location has been added in Table 2 and Fig. 1. The rs2290602 SNP had the lowest *p *value in the dominant mode (*p *= 0.00095, Table 2), and rs2290602 was significantly associated with NAFLD even when the most conservative Bonferroni's correction was applied (*p *= 0.00095 × 15 × 3 = 0.04275). The OR (95% CI) was 2.73 (1.48 – 5.06) in the dominant mode, and thus the relative risk of developing NAFLD of the subjects with the T allele was 2.73 fold higher than among the subjects without the T allele. All SNPs except rs2290602 were in Hardy-Weinberg equilibrium (*p *> 0.1). The departure of rs2290602 from the Hardy-Weinberg equilibrium was detected in the NAFLD group (*p *= 0.02), because this SNP is associated with NAFLD and the cases were selected for the phenotype. Case may be biased compared to the general population. Thus, it is not unexpected that case was not in Hardy-Weinberg equilibrium. Linkage disequilibrium (LD) analysis revealed the presence of two blocks in the *PPARG1A *gene. SNP rs2290602 and 11 other SNPs (rs2290604, rs3774907, rs3774908, rs2290603, rs2970849, rs2932968, rs3755863, rs3736265, rs3774920, rs768695, and rs3774923) were in the same block (Fig. 1).

**Table 1:** Characteristics of the NAFLD patients and control subjects.

	Control	NAFLD	*P*-value
Men/Women	112/329	51/64	
Age (years)	47.4 ± 15.9	49.6 ± 15.1	0.20
BMI (kg/m^2^)	21.2 ± 2.2	27.4 ± 5.3	<0.0001
FBS (mg/dL)	90.4 ± 7.4	117.2 ± 32.0	<0.0001
HbA1c (%)	5.1 ± 0.6	5.8 ± 1.3	<0.0001
Total Cholesterol (mg/dL)	200.9 ± 33.4	211.7 ± 36.5	0.0027
Triglycerides (mg/dL)	72.2 ± 27.6	168.9 ± 110.7	<0.0001
HDL cholesterol (mg/dL)	68.1 ± 13.9	52.9 ± 20.9	<0.0001
Systolic blood pressure (mmHg)	110.5 ± 9.9	124.1 ± 14.8	<0.0001
Diastolic blood pressure (mmHg)	69.2 ± 7.3	76.8 ± 11.3	<0.0001
AST (IU/L)	20.1 ± 6.3	44.4 ± 26.3	<0.0001
ALT (IU/L)	15.7 ± 6.5	70.4 ± 53.6	<0.0001

Data are means ± SD.

**Table 2:** Genotype frequencies and association tests of SNPs PPARGC1A gene using NAFLD and control

Rs number	Location	allele1/allele2	NAFLD			Control			Allele frequency mode			Recessive mode			Dominant mode		
			
			11	12	22	11	12	22	χ^2^	*P*-value	OR ^a ^(95% CI)	χ^2^	*P*-value	OR^a ^(95% CI)	χ^2^	*P*-value	OR^a ^(95% CI)
rs3774902	Intron 1 +745	T/C	46	49	19	207	170	63	1.6	0.2008	0.82 (0.61 – 1.11)	1.6	0.2010	0.76 (0.50 – 1.16)	0.4	0.52917	0.84 (0.48 – 1.46)
rs2970871	Intron 1 +945	A/G	31	64	19	121	212	107	1.0	0.3225	1.16 (0.87 – 1.55)	0.0	0.9478	0.98 (0.62 – 1.56)	3.0	0.08241	1.61 (0.94 – 2.75)
rs2970876	Intron 1 +2908	C/T	38	55	22	109	226	102	2.8	0.0964	1.28 (0.96 – 1.72)	3.1	0.0804	1.49 (0.95 – 2.32)	0.9	0.33573	1.29 (0.77 – 2.15)
rs2290604	Intron 4 +848	A/G	77	33	3	261	159	21	3.2	0.0716	1.42 (0.97 – 2.07)	3.0	0.0815	1.48 (0.95 – 2.29)	1.0	0.32629	1.83 (0.54 – 6.26)
rs3774907	Intron 5 +161	T/C	75	35	3	280	138	21	0.6	0.4292	1.16 (0.80 – 1.69)	0.3	0.6083	1.12 (0.72 – 1.73)	1.0	0.32239	1.84 (0.54 – 6.29)
rs3774908	Intron 5 +1089	T/C	79	33	3	261	158	21	3.6	0.0580	1.44 (0.99 – 2.10)	3.4	0.0661	1.51 (0.97 – 2.33)	1.0	0.30970	1.87 (0.55 – 6.39)
rs2290603	Intron 5 +3590	A/G	75	35	4	264	154	19	1.0	0.3075	1.21 (0.84 – 1.75)	1.1	0.2933	1.26 (0.82 – 1.94)	0.2	0.68996	1.25 (0.42 – 3.75)
rs2290602	Intron 7 +171	T/G	34	67	13	99	222	113	8.5	0.0036	1.55 (1.15 – 2.08)	2.4	0.1201	1.44 (0.91 – 2.28)	10.9	0.00095	2.73 (1.48 – 5.06)
rs2970849	Intron 7 +8096	A/G	68	42	5	232	173	34	2.2	0.1385	1.30 (0.92 – 1.82)	1.4	0.2287	1.29 (0.85 – 1.96)	1.6	0.20492	1.85 (0.71 – 4.83)
rs2932968	Intron 7 +8494	T/C	74	36	4	262	157	21	1.2	0.2809	1.22 (0.85 – 1.76)	1.1	0.2959	1.26 (0.82 – 1.93)	0.3	0.56233	1.38 (0.46 – 4.10)
rs3755863	Exon 8 +707 (T/T)	G/A	32	60	23	82	222	135	7.3	0.0070	1.49 (1.11 – 2.00)	4.7	0.0308	1.68 (1.05 – 2.69)	5.2	0.02301	1.78 (1.08 – 2.93)
rs3736265	Exon 9 +42 (M/T)	A/G	81	30	3	261	158	20	5.0	0.0259	1.55 (1.05 – 2.29)	5.2	0.0231	1.67 (1.07 – 2.62)	0.8	0.35922	1.77 (0.52 – 6.05)
rs3774920	Intron 10 +1732	A/G	79	32	3	263	157	21	3.8	0.0528	1.46 (0.99 – 2.14)	3.6	0.0587	1.53 (0.98 – 2.37)	1.0	0.31886	1.85 (0.54 – 6.31)
rs768695	Intron 12 +4535	T/C	69	35	9	229	184	27	1.2	0.2737	1.21 (0.86 – 1.70)	2.9	0.0863	1.44 (0.95 – 2.20)	0.5	0.48225	0.76 (0.34 – 1.66)
rs3774923	Exon 13 +2485	A/G	82	30	3	263	157	20	5.0	0.0256	1.55 (1.05 – 2.29)	5.2	0.0232	1.67 (1.07 – 2.61)	0.9	0.35349	1.78 (0.52 – 6.09)

^a ^OR; odds ratio,

Next, we divided the NAFLD group into the NASH and the steatosis group and used rs2290602 to perform case-control association studies. Although it was not conclusive because of the small number of data, the frequency of T allele was significantly higher in the NASH group than in the control group (*p *= 0.00093, allele frequency mode). When the frequency of the T allele in the NASH group was compared to its frequency in the steatosis group, its frequency was found to tend to be higher than in the steatosis group (*p *= 0.09). Thus, rs2290602 was significantly more closely associated with NASH than with simple steatosis (Table 3).

**Table 3:** Results of testing for differences in SNP rs2290602 frequency between NAFLD group and control group, NASH group and contol group, NASH group and simple steatosis group.

	NAFLD			Control			Allele frequency mode			Recessive mode			Dominant mode		
	
Study	11	12	22	11	12	22	χ^2^	*P*-value	Odds ratio (95% CI)	χ^2^	*P*-value	Odds ratio (95% CI)	χ^2^	*P*-value	Odds ratio (95% CI)
NAFLD vs. Control	34	67	13	99	222	113	1.6	0.00363	1.55 (1.15 – 2.08)	2.4	0.12008	1.44 (0.91 – 2.28)	10.9	0.00095	2.73 (1.48 – 5.06)
NASH vs. Control	24	34	6	99	222	113	1.0	0.00093	1.90 (1.29 – 2.79)	6.5	0.01097	2.03 (1.17 – 3.53)	8.5	0.00352	3.40 (1.43 – 8.10)
NASH vs. simple steatosis	24	34	6	10	33	7	2.8	0.09168	1.58 (0.93 – 2.69)	4.1	0.04270	2.40 (1.02 – 5.66)	0.6	0.44076	1.57 (0.49 – 5.02)

To investigate whether the genotype of rs2290602 SNP were associated with the clinical parameters, we compared age, BMI, and the fasting plasma glucose, immunoreacrive insulin, HbA1c, total cholesterol, triglyceride, HDL cholesterol, AST, ALT, VFA, and SFA between the NAFLD patients with different genotypes (GG vs. GT + TT, or TT vs. GT + GG) by Student's *t*-test. The results showed that serum AST and ALT values of the NAFLD patients with the TT allele were significantly higher than those of the NAFLD patients with the GT or GG allele at SNP rs2290602 (Table 4). When the subjects were limited to the NASH patients, the serum AST and ALT values were also significantly higher in the TT genotype group than in the GT group or GG group at SNP rs2290602 (*p *= 0.0177, *p *= 0.0140, respectively).

**Table 4:** Comparison between various quantitative phenotypes in groups of NAFLD patients with different SNP genotypes at rs2290602

	GG	GT+TT	p value	GG+GT	TT	p value
N	14	101		80	35	
Age (years)	48.5 + 18.5	49.5 + 14.7	0.8303	48.4 + 14.4	51.7 + 16.4	0.2913
BMI (kg/m2)	27.3 + 7.2	27.2 + 4.8	0.9194	27.0 + 5.1	27.6 + 4.9	0.5581
FBS (mg/dl)	118.5 + 26.3	117.3 + 32.9	0.8929	116.8 + 32.0	118.7 + 32.8	0.7791
IRI (ul/ml)	13.6 + 6.2	13.7 + 8.9	0.9778	12.9 + 6.9	15.4 + 11.6	0.1865
HbA1c (%)	5.39 + 1.26	5.88 + 1.26	0.1890	5.83 + 1.28	5.80 + 1.23	0.9138
Total cholesterol (mg/dl)	219.1 + 35.7	213.1 + 39.3	0.6017	214.7 + 39.5	211.6 + 37.5	0.6978
HDL cholesterol (mg/dl)	50.6 + 10.0	53.3 + 22.0	0.6709	51.2 + 16.2	57.0 + 29.1	0.1800
Triglycerides (mg/dl)	144.9 + 53.7	172.1 + 116.4	0.4102	159.1 + 70.4	192.2 + 172.3	0.1469
AST (U/ml)	35.0 + 12.2	45.8 + 27.5	0.1660	40.4 + 21.3	54.5 + 34.1	0.0085
ALT (U/ml)	50.4 + 27.2	73.4 + 55.7	0.1462	62.7 + 37.7	89.9 + 76.9	0.0127
VFA (cm2)	129.3 + 32.9	135.8 + 50.0	0.7585	135.4 + 47.4	134.9 + 51.3	0.9703
SFA (cm2)	274.6 + 148.9	229.4 + 116.8	0.3821	235.4 + 123.8	230.4 + 114.2	0.8709

Data are shown as means ± SD. VFA: visceral fat area, SFA: subcutaneous fat area, AST: aspartate aminotransferase, ALT: alanine aminotransferase, FBS: fasting blood sugar, IRI: Immunoreactive insulin,

### mRNA expression of PPARGC1A in the liver

**Figure 2:**
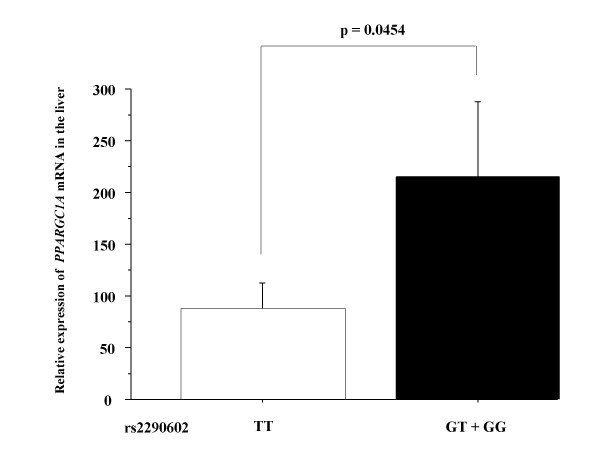
**The expression of mRNA for *PPARGC1A *in the liver**. Significant difference in intrahepatic mRNA expression of *PPARGC1A *between the TT group (n = 8) and the GG or GT group (n = 10) at SNP rs2290602 (p = 0.0454).

Expression of *PPARGC1A *mRNA in the liver biopsy specimens of NAFLD patients was evaluated by real-time RT-PCR. The results obtained by real-time RT-PCR in the GG or GT group (n = 10) were compared with the results in the TT group (n = 8) at SNP rs2290602. As shown in Fig. 2, the expression of *PPARGC1A *mRNA transcripts in the liver was significantly lower in the TT group than in the GG or GT group (p = 0.0454).

## Discussion

Genetic factors are important to the development of NAFLD, and recent advantages in SNP genotyping methods have enabled the detection of genetic the variations associated with increased susceptibility to NAFLD. There have been a few reports of genetic variations that are associated with NAFLD, and they have been in the genes for TNF receptor 2 (TNFR-2), TNF-α, micorosomal triglyceride transfer protein (MTP), and methylenetetrahydrofolate reductase (MTHFR) genes [6,7,26]. All of these genes are related to inflammation, lipid metabolism, and oxidation. We examined the 15 SNPs in *PPARGC1A *and found that rs2290602 was significantly associated with NAFLD, especially with NASH, and the frequency of the T allele of rs2290602 was significantly higher in the NASH patients than in the control subjects. We also found that intrahepatic *PPARGC1A *mRNA expression was significantly lower in the TT group than in the GG or GT group at SNP rs2290602.

*PPARGC1A *interacts with peroxisome proliferators-activated receptors (PPARs) and has various functions, including as integrator of the molecular regulatory circuitry involved in the transcriptional control of cellular energy metabolism, including mitochondrial function and biogenesis [27,28], and regulation of gene expression involved in lipid and glucose metabolism. The *PPARGC1A *Gly482Ser polymorphism is also associated with obesity, hypertension, and diabetes [14-18,29]. NAFLD is often accompanied by obesity, hypertriglyceridemia, type 2 diabetes, and insulin resistance [30], and *PPARGC1A *knockout mice develop hepatic steatosis [12]. Thus, variations in rs2290602 in the *PPARGC1A *gene would be expected to affect lipid and glucose metabolism, and result in the development of NAFLD and NASH.

Recent evidence also implicates *PPARGC1A *in the homeostatic control of systemic energy metabolism, and *PPARGC1A *has been shown to regulate several key hepatic gluconeogenic genes [31-34]. Recent studies have also shown altered expression of *PPARGC1A *and downstream mitochondrial target pathways in the skeletal muscle of humans with insulin resistance and diabetes [35-37].

This study had a limitation in regard to the control subjects. The number of the patients was too small for a polymorphism study. Although none of the control subjects had abnormal liver function or was obese, we cannot completely rule out the possibility that the control group included patients with mild steatosis, since we did not perform liver biopsies in the control subjects.

## Conclusion

In conclusion, this is the first study to demonstrate a significant association between genetic variations in *PPARGC1A *and NAFLD. This finding suggested that *PPARGC1A *polymorphism and lower expression of *PPARGC1A *mRNA in the liver are an important genetic contribution to the etiology of NAFLD.

## Abbreviations

BMI: body mass index; NAFLD: nonalcoholic fatty liver disease; NASH: nonalcoholic steatohepatitis; SNPs: single nucleotide polymorphisms; VFA: visceral fat area; SFA: subcutaneous fat area; AST: aspartate aminotransferase; ALT: alanine aminotransferase.

## Competing interests

The authors declare that they have no competing interests.

## Authors' contributions

MY performed the literature review, collected the clinical data, and drafted the manuscript, with contributions from YN, HE, and KH. KF and KH organized the field survey for data collection. SK, HM, KY, HT, HK, MI, YA, KK, SS, and WK collected the clinical data. SM analyzed the liver pathology. HI, KH, TU, and NK revised the manuscript. AN was responsible for the design of the study. All authors read and approved the final manuscript.

## Pre-publication history

The pre-publication history for this paper can be accessed here:


